# Spontaneous idiopathic pneumoperitoneum presenting as an acute abdomen: a case report

**DOI:** 10.1186/1752-1947-5-86

**Published:** 2011-02-27

**Authors:** Michail Pitiakoudis, Petros Zezos, Anastasia Oikonomou, Michail Kirmanidis, Georgios Kouklakis, Constantinos Simopoulos

**Affiliations:** 1 Second Department of Surgery, Democritus University of Thrace, University General Hospital, 68100 Dragana Alexandroupolis, Greece; 2 Gastrointestinal Endoscopy Unit, Democritus University of Thrace, University General Hospital, 68100 Dragana Alexandroupolis, Greece; 3 Radiology Department, Democritus University of Thrace, University General Hospital, 68100 Dragana Alexandroupolis, Greece

## Abstract

**Introduction:**

Pneumoperitoneum is most commonly the result of a visceral perforation and usually presents with signs of acute peritonitis requiring an urgent surgical intervention. Non-surgical spontaneous pneumoperitoneum (not associated with a perforated viscus) is an uncommon entity related to intrathoracic, intra-abdominal, gynecologic, iatrogenic and other miscellaneous causes, and is usually managed conservatively. Idiopathic spontaneous pneumoperitoneum is an even more rare condition from which both perforation of an intra-abdominal viscus and other known causes of free intraperitoneal gas have been excluded.

**Case presentation:**

We present the case of an idiopathic spontaneous pneumoperitoneum. A 69-year-old Greek woman presented with acute abdominal pain, fever and vomiting. Diffuse abdominal tenderness on deep palpation without any other signs of peritonitis was found during physical examination, and laboratory investigations revealed leukocytosis and intraperitoneal air below the diaphragm bilaterally. Her medical history was unremarkable except for previous cholecystectomy and appendectomy. The patient did not take any medication, and she was not a smoker or an alcohol consumer. Emergency laparotomy was performed, but no identifiable cause was found. A remarkable improvement was noticed, and the patient was discharged on the seventh postoperative day, although the cause of pneumoperitoneum remained obscure.

**Conclusion:**

A thorough history and physical examination combined with the appropriate laboratory tests and radiologic techniques are useful tools in identifying patients with non-surgical pneumoperitoneum and avoiding unnecessary operations.

## Introduction

Pneumoperitoneum is the result of a gastrointestinal (GI) tract perforation in more than 90% of cases [1]. Perforation of the stomach or duodenum caused by peptic ulcer is considered the most common cause of pneumoperitoneum. Pneumoperitoneum can also be the result of a diverticular rupture or of an abdominal trauma [1]. It commonly presents with signs and symptoms of peritonitis, and subphrenic free gas in an upright chest radiograph is the most common radiologic finding. In most cases, pneumoperitoneum requires urgent surgical exploration and intervention [1].

However, sometimes pneumoperitoneum not associated with a perforated viscus can occur; this is called spontaneous pneumoperitoneum (SP) or "non-surgical" pneumoperitoneum. SP is associated with intrathoracic, intraabdominal, gynecologic, iatrogenic or other miscellaneous causes [1]. Although it is not usually complicated with peritonitis, SP is characterized by a benign course and can be managed conservatively [1-4]. Idiopathic SP is an even more rare condition for which no clear etiology has been established because both perforation of an intraabdominal viscus and other known causes of free intraperitoneal gas have been excluded [1,5-7]. Idiopathic pneumoperitoneum is usually diagnosed after negative laparotomy results. SP poses significant management dilemmas for surgeons, especially when signs of peritonitis are absent or when the cause is unknown before laparotomy.

## Case presentation

A 69-year-old Greek female patient presented at our emergency department (ED) with a two-hour history of abdominal pain and vomiting. Her medical history was unremarkable except for previous cholecystectomy and appendectomy. The patient did not take any medications, and she was not a smoker or an alcohol consumer.

**Figure 1:**
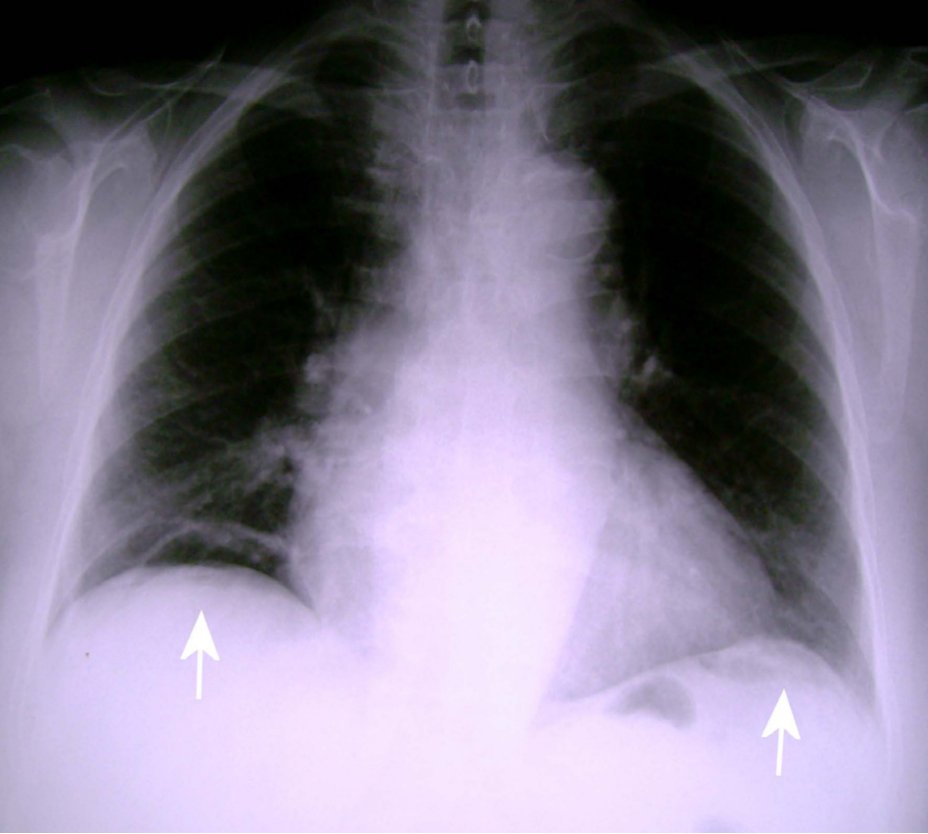
**Upright posteroanterior chest radiograph**. There is free subdiaphragmatic air bilaterally that is more clearly noted on the right side (*white arrows*).

**Figure 2:**
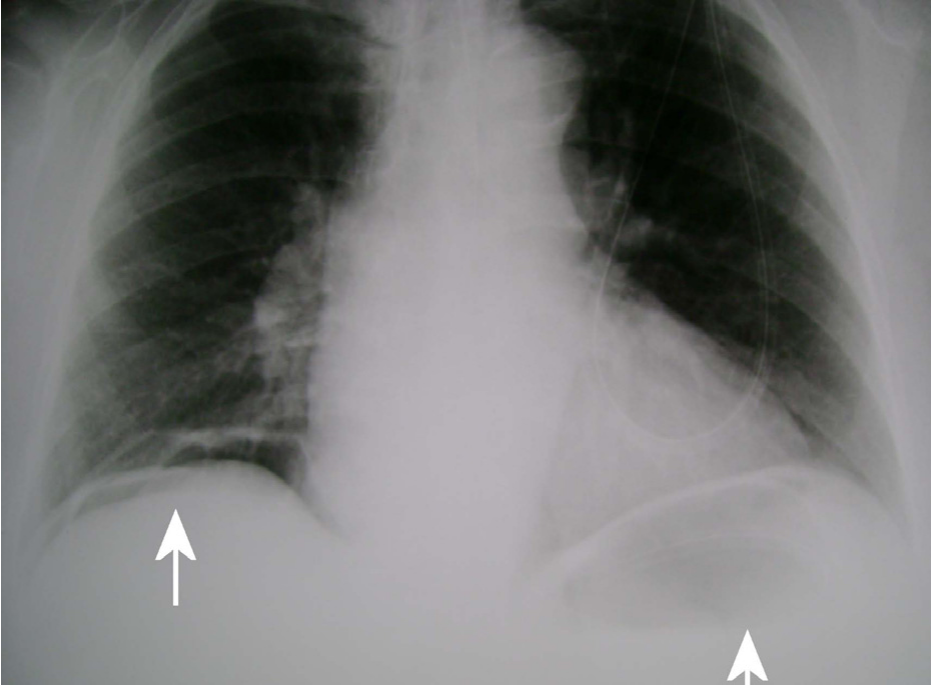
**Upright posteroanterior chest radiograph after insufflating air into the stomach**. The free subdiaphragmatic air has slightly increased in size bilaterally compared with Figure 1 (*white arrows*).

She looked ill with a blood pressure of 130/85 mm/Hg, a pulse rate of 90 beats/min, respirations of 25 breaths/min and a temperature of 38.5°C. A thorough physical examination revealed diffuse abdominal tenderness on deep palpation without any other signs of peritonitis. The laboratory examination was unremarkable except for polymorphonuclear leucocytosis (white blood cell [WBC] count, 15 × 10^3^/μL; neutrophils, 86%) and an elevated C-reactive protein (14 mg/dL; reference range, 0-5). An upright chest radiograph demonstrated free subdiaphragmatic air bilaterally (Figure 1), which seemed to be increasing during air insufflation in the stomach via a nasogastric tube (Figure 2). Abdominal ultrasound examination was unremarkable.

An emergency laparotomy was performed for a suspected perforation in the upper GI tract. A few adhesions caused by previous cholecystectomy and appendicectomy were observed without any signs of peritoneal irritation or peritoneal fluid. The stomach and duodenum were fully mobilized, and the lesser sac was explored, but no evidence of perforation was found in the distal esophagus, stomach or duodenum. The small bowel and colon were also examined, but no leakage was observed. Subsequently, dilution of methylene blue in normal saline was instilled into the stomach through the nasogastric tube, but no obvious leakage was noted. Afterward, the abdominal cavity was filled with 2000 cc of normal saline, and air was again infused through the nasogastric tube into the stomach, but no air leakage from the upper GI tract was noted. Finally, because no cause of the pneumoperitoneum had been found, the operation was completed by placing a double-lumen drain.

**Figure 3:**
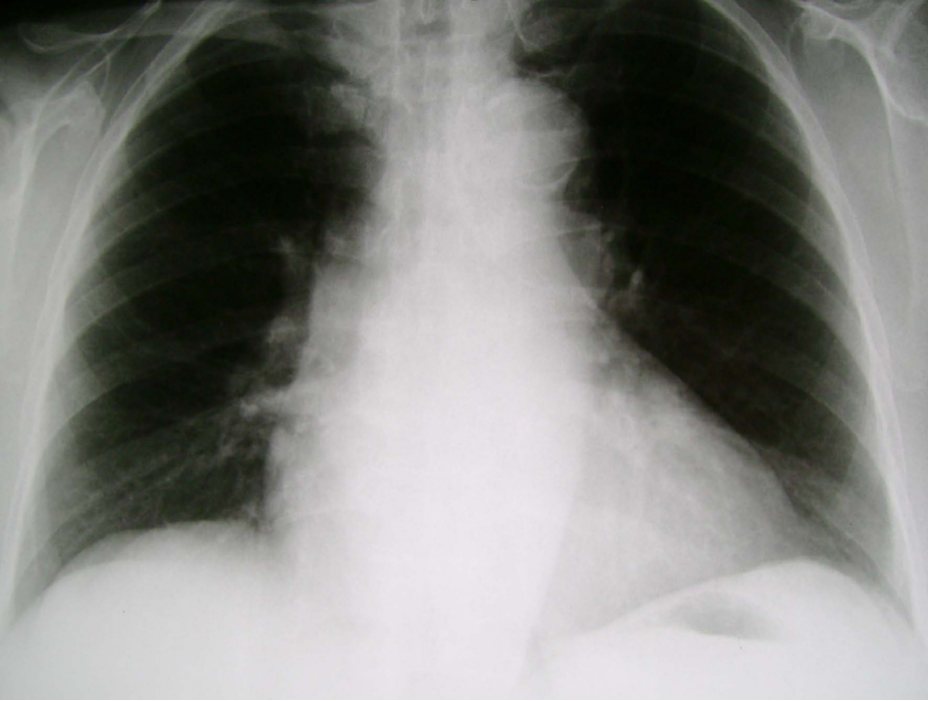
**Upright posteroanterior chest radiograph just before the patient's discharge**. No subdiaphragmatic free air is noted bilaterally.

The postoperative course was uneventful, and the patient showed a significant and prompt recovery. The subdiaphragmatic air disappeared six days postoperatively (Figure 3). The patient was discharged home on the seventh postoperative day. One month later, esophagogastroduodenoscopy, colonoscopy and abdominal computed tomography (CT) were performed, but no pathology was detected.

## Discussion

SP is associated with intrathoracic, intraabdominal, gynecologic, iatrogenic and other miscellaneous causes [1,2]. SP has been attributed to several thoracic causes, such as traumas (including barotraumas), pneumothorax and bronchoperitoneal fistulas [1]. SP can be accompanied by pneumomediastinum or pneumopericardium, especially in patients who are on mechanical aspiration and positive end-expiration pressure [1]. In extremely rare cases, scuba diving and pulmonary sepsis can cause SP. Pneumatosis cystoides intestinalis is the most common abdominal cause of nonsurgical pneumoperitoneum [1]. Emphysematous cholecystitis, spontaneous bacterial peritonitis, ruptured hepatic abscess and perforated pyometra in women are rare causes of SP [1]. In women, pneumoperitoneum after rough sexual intercourse or after Jacuzzi usage has also been reported because the air can also be transmitted to the peritoneal cavity through the vagina and saplings [1]. Laparoscopic or endoscopic procedures (colonoscopy) may cause iatrogenic SP [1].

The cause of pneumoperitoneum and the clinical signs determine its mode of treatment, surgical or not. When signs and symptoms of "acute abdomen" are present, surgical management is mandatory, but in cases of nonsurgical pneumoperitoneum with mild symptoms and without any signs of peritonitis, conservative treatment is indicated [2].

A detailed history and physical examination can be very helpful in distinguishing surgical from nonsurgical pneumoperitoneum, thus avoiding unnecessary laparotomies [2]. Moreover, radiographic imaging before and after air insufflation into the gastric lumen via a nasogastric tube (pneumogastrogram) is an easy and safe method, which can enhance or confirm the diagnosis of a visceral perforation in the upper GI tract [8].

Plain chest or abdominal radiography is the most common imaging examination for the diagnosis of even very small amounts of intraperitoneal free air in the ED setting [9], but abdominal CT is a more sensitive method of diagnosing pneumoperitoneum and identifying the cause of "acute abdomen" [10,11]. Moreover, modern technology with multidetector CT is highly accurate for predicting the site of GI tract perforations [12,13].

It has been proposed that in some cases with idiopathic pneumoperitoneum, a subclinical small visceral perforation may have occurred, permitting only the leakage of air and not of bowel contents [1]. Finally, in other cases, other unknown factors may be the cause of idiopathic pneumoperitoneum [1].

We report the case of a patient who underwent an urgent but nondiagnostic exploratory laparotomy, although she had compelling evidence for a surgical pneumoperitoneum. A minority of pneumoperitoneum cases are considered idiopathic, but many of them undergo surgical exploration [2]. van Gelder *et al. *[5] reported six patients with pneumoperitoneum and clinical signs of acute abdomen who underwent exploratory laparotomy, which did not reveal any intraabdominal pathology. Chandler *et a*l. [14] reported a laparotomy rate of 28% on nonsurgical pneumoperitoneum. In a review, Mularski *et al. *[15] found 196 reported cases of nonsurgical pneumoperitoneum, of which 45 underwent surgical exploration without evidence of perforated viscus. Furthermore, Mularski *et al. *[15] reported that 11 of 36 (31%) miscellaneous or idiopathic cases of nonsurgical PP underwent surgical exploration.

Currently, laparoscopic exploration instead of laparotomy can be the operation of choice in cases of pneumoperitoneum because it can both determine and treat the cause, offering all the advantages of minimally invasive surgery.

## Conclusion

A thorough history and physical examination combined with the appropriate laboratory tests and radiologic techniques are useful tools in identifying patients with nonsurgical pneumoperitoneum and avoiding unnecessary operations.

## List of abbreviations

CT: computed tomography; ED: emergency department; GI: gastrointestinal; SP: spontaneous pneumoperitoneum; WBC: white blood cell.

## Consent

Written informed consent was obtained from the patient for the publication of this case report and the accompanying images. A copy of the written consent is available for review by the Editor-in-Chief of this journal.

## Competing interests

The authors declare that they have no competing interests.

## Authors' contributions

MP participated in the patient's treatment, had the idea for the case report, contributed to the first draft and performed all of the revisions. PZ collected the patient's data, participated in the first draft and performed all of the revisions. AO participated in the imaging diagnosis of the case and contributed to the writing of the paper. MK participated in the patient's treatment and contributed to the writing of the paper. GK contributed to the writing of the paper. CS participated in the patient's treatment and participated in the final revision. All authors read and approved the final manuscript.
